# COVID‐19, Virchow's triad and thromboembolic risk in obese pregnant women

**DOI:** 10.1002/clc.23602

**Published:** 2021-03-24

**Authors:** Lionel Carbillon, Marion Fermaut, Amelie Benbara, Jeremy Boujenah

**Affiliations:** ^1^ Hôpital Jean Verdier, Assistance Publique—Hôpitaux de Paris Bondy France; ^2^ Université Sorbonne Paris Nord Bobigny France

To the Editor

Overweight and obesity in pregnant women are risk factors for both thromboembolism^1^ and severe COVID‐19,^2^ and a body of evidence indicates that the benefit–risk assessment is in favor of thromboprophylaxis, as soon as the diagnosis of Coronavirus‐Infectious‐Disease‐2019 (COVID‐19) has been made in these patients.

Indeed, from their recent reviews analyzing the three pivotal factors described by Virchow in the genesis of thromboembolism (TE), Ahmed et al^2^ as well as Mehta et al,^3^ showed that all components of this classic triad contributed together to an increased thromboembolic risk during the course of COVID‐19. Thus, angiotensin‐converting enzyme 2 (ACE2) protein, which constitutes the functional receptor for SARS coronavirus, is present in endothelial cells, thus allowing the virus to enter and to induce endothelial dysfunction.^2^, ^3^ In addition, hypercoagulability occurs early in the disease due to an overwhelming inflammatory state. Lastly, blood flow is altered by the elevated fibrinogen which is a major determinant of blood viscosity, and of course by blood stasis when the patient is hospitalized and immobilized.

Importantly, a similar analysis of the questions related to the pathophysiology of thromboembolism during pregnancy and the peripartum period, thanks to these three categories of contributing factors characterized by Virchow, has also been applied to the high risk of TE as pregnancy progresses.^4^ Indeed, firstly, the gravid uterus mechanically compresses veins as its volume increases, while the action of progesterone induces a loss of tone of the vein wall,^4^ and these factors combine their effect to slow blood flow. Secondly, a state of hypercoagulability culminates in late gestation and is partly explained by, and well reflected in the highly significant rise in d‐dimer levels from the first to the third trimester.^5^ In this context, any factor shifting the “hemostasis imbalance” is associated with an increased risk of maternal TE. Lastly, during pregnancy circulating cytokines and growth factors may contribute to the breakdown of the endothelial monolayer.^4^ In this regard, obese pregnancy is associated with chronic preexisting impairment of endothelial function secondary to even increased production of inflammatory T‐helper cells‐2 cytokines.^6^ Thus, the increased thromboembolic risk related to the gestational state has particularly been evidenced in obese pregnant women, and continues for up to 6–12 weeks postpartum.^1^ Moreover, the adjusted odds of antepartum and postpartum venous TE increases progressively with increasing BMI,^1^ with obesity class III women having the highest risk of pregnancy‐related venous TE compared with normal BMI women. And precisely, obesity, which affects an increasing proportion of pregnant women, is both a risk factor for TE during pregnancy, and a risk factor for COVID‐19 progression, in the event of SARS‐COV‐2 infection. In a UK national population based cohort study including all pregnant women admitted to hospital with confirmed SARS‐CoV‐2 infection between March 1, 2020, and April 14, 2020, 70% of pregnant women admitted to hospital were overweight or obese.^7^ In another USA multi‐center cohort study describing the clinical course of COVID‐19 in pregnant women admitted to the hospital for the treatment of severe and/or critical disease, the mean BMI was 33.5 kg/m^2^.^8^

A body of evidence supports that an inflammatory coagulopathy occurs early in the disease due to an overwhelming “inflammatory state.”^9^, ^10^ Tang et al^11^ showed that in a cohort of 449patients with severe COVID‐19 and markedly elevated d‐dimer (d‐dimer >6‐fold of the upper limit of normal), the 28‐day mortality of heparin users was lower than that of the non‐users (32.8% vs. 52.4%, *p* = .017). Similarly, in their multi‐center cohort study describing the clinical course of COVID‐19 in pregnant women admitted to the hospital for the treatment of severe or critical disease, Pierce‐Williams et al^8^ reported that 57% and 5% of the cases received prophylactic and therapeutic heparin/ low‐molecular‐weight heparin (LMWH), respectively. Even more importantly, these authors indicated that the need for therapeutic heparin/LMWH was significantly associated with critical condition (*p* < .001).

Both the International Subcommittee for Women's Health Issues in Thrombosis and Haemostasis (ISTH) and the American Society of Hematology recommended that all hospitalized COVID‐19 patients receive thromboprophylaxis.^12^ Moreover, the ISTH recommended that prophylaxis with LMWH should be considered “in the presence of immobility, high fever, dehydration, or additional maternal risk factors for venous TE”.^12^

However, as the available data show that overweight/obesity in pregnant women is a strong risk factor for both thromboembolism and severe COVID‐19, the benefit–risk assessment is in favor of anticoagulant prophylaxis, as soon as the diagnosis of COVID‐19 has been made in these patients, and it appears sound to recommend this practice. Unfractionated heparin and LMWH do not cross the placenta, are safe for the fetus,^13^ and LMWH is the drug of choice for the prevention and treatment of venous TE during pregnancy because of its practical advantages over heparin and unfractionated heparin, lower risk of side effects, and safety profile.^13^

In critically ill pregnant patients, a caesarean section (with its bleeding risk) may be indicated at any time if the maternal condition worsens. Thus, each patient deserves individualized assessment of her specific risk profile, justifying that the management is multidisciplinary and the dose of anticoagulants be adjusted on a case‐by‐case basis.

## CONFLICT OF INTEREST

The authors declare no potential conflict of interest.

## AUTHOR CONTRIBUTIONS

Lionel Carbillon: Conception and design; Lionel Carbillon, Marion Fermaut, Amelie Benbara, Jeremy Boujenah: Analysis and interpretation of literature; Lionel Carbillon: Drafting of manuscript.
